# Stage IV EGFR Mutation-Negative and ALK Mutation-Negative Lung Adenocarcinoma: Long-Term Survival is Possible

**DOI:** 10.7759/cureus.419

**Published:** 2015-12-19

**Authors:** Shiksha Kedia, Gwenalyn Garcia, Meekoo Dhar

**Affiliations:** 1 Department of Hematology and Oncology, Staten Island University Hospital

**Keywords:** lung cancer, stage iv lung cancer, long-term survival

## Abstract

Lung cancer is the leading cause of cancer death in the United States with a five-year survival of 16.8% for all stages and median survival of four months for Stage IV disease. We report a case of a 54-year-old male with a seven-year survival after being diagnosed with Stage IV epidermal growth factor receptor (EGFR) mutation-negative and anaplastic lymphoma kinase (ALK) mutation-negative adenocarcinoma of the lung, demonstrating an exceptional response to treatment.

## Introduction

Lung cancer is the leading cause of cancer death in the United States. In 2015, an estimated 221,200 new cases (115,610 men and 105,590 women) of lung and bronchial cancer will be diagnosed, and 158,040 deaths (86,380 men and 71,660 women) are estimated to occur because of the disease [1]. About 16.8% of all patients with lung cancer are alive at five years after diagnosis [2]. According to the US Surveillance, Epidemiology, and End Results database, median survival is only four months for Stage IV disease. Disease with malignant pleural effusion had a five-year survival of only 2% [3]. We report a case of a 54-year-old male with a seven-year survival after being diagnosed with Stage IV adenocarcinoma of the lung.

## Case presentation

A 48-year-old male ex-smoker was diagnosed with lung cancer in November 2008. His Eastern Cooperative Oncology Group (ECOG) performance status (PS) was 0. Family history was significant for lung cancer in his maternal grandmother, breast cancer in his paternal grandmother, and brain cancer in his mother.

He initially presented with left-sided chest pain. A computed tomography (CT) scan of the chest revealed locally advanced disease involving the left hemithorax with associated pleural effusion and pleural thickening. A positron emission tomography (PET) scan showed circumferential enhancing nodular thickening of the entire left pleura with extension into the interlobar fissure. The left pleural effusion demonstrated mild hypermetabolic activity with standardized uptake value (SUV) between 1.7 to 2.5. No mediastinal or hilar nodes were noted. Informed patient consent was obtained for treatment. 

The patient underwent wedge resection of the left upper and lower lobe and bilateral pleural biopsies in March 2009. The pathology showed poorly differentiated carcinoma. The largest focus of the tumor measured 0.7 cm, with positive margins involving the lung parenchyma, subpleural surface, and lymphovascular space. Immunohistochemistry was positive for TTF-1, CK-7, and CEA. There were focal rare cells positive for p63. The tumor was negative for CK-20, calretinin, WT-1, and CK-5/6. This was consistent with primary adenocarcinoma of the lung. Due to the pleural involvement and absence of extrathoracic metastatic disease, he was staged as Stage IV, M1a. EGFR and ALK mutational analysis were not performed as this was not routinely recommended at that time. 

A repeat PET scan in April 2009, prior to the start of chemoradiation (CRT), showed stable disease without any involvement of new sites.  He underwent radiation therapy (RT) to the left lung and pleural space from May through July, with 1.8 Gy per day for 33 fractions to a total dose of 59.4 Gy using a 6 MV photon beam and 8-field CT-based intensity-modulated radiation therapy. Because of the large field size, concurrent CRT was not given. He then received chemotherapy with carboplatin on day 1 and docetaxel on days 1 and 8 of a 21-day cycle for four cycles. His course was complicated by a pulmonary embolism, and he was started on appropriate anticoagulation.

**Figure 1:**
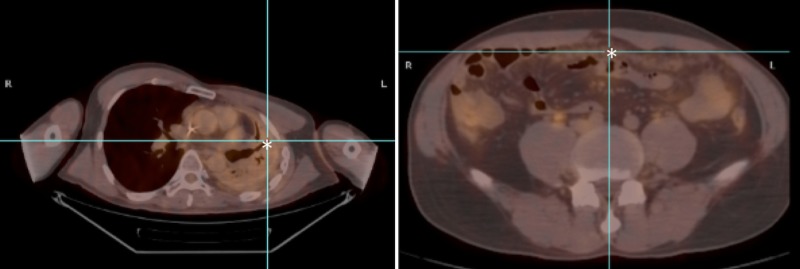
PET scan showing stable abnormal activity within the left lung, likely reflecting post-treatment change (left), and new omental nodularity (right), indicating disease progression.

After completion of chemotherapy, erlotinib, 100 mg by mouth daily, was given as maintenance therapy. In July 2010, a repeat PET scan showed stable left lung disease and stable pleural disease within the left hemithorax. However, new non-fludeoxyglucose (FDG)-avid nodularity within the anterior omentum and new abdominopelvic ascites were noted (Figure 1). Adenocarcinoma of the lung was subsequently confirmed on laparoscopic omental biopsy. The biopsy specimen later tested negative for both EGFR mutation and ALK rearrangement.

Treatment was switched to pemetrexed and bevacizumab. Follow-up scans showed a good response with a decrease in size and extent of the omental carcinomatosis. He no longer required treatment for pain with narcotics. He continued to be on this regimen from October 2011 to February 2013. His course was complicated by hematuria of unclear etiology, later attributed to warfarin use. This required treatment with bevacizumab to be held intermittently. 

On subsequent imaging, new liver lesions and sigmoid thickening were noted. The liver lesions were too small to be biopsied. Colonoscopy and biopsy of the involved site were negative for malignancy. He was then switched to a regimen of carboplatin, paclitaxel, and bevacizumab, which he received every three weeks for six cycles. He completed treatment in June 2013 and remained clinically stable off treatment for two years. A PET scan done in July 2015 showed stable disease in the left lung and non-FDG avid omental nodularity and abdominal ascites.

In October 2015, during outpatient follow-up, he expressed concern regarding prolonged use of the warfarin and wished to come off of the anticoagulation. After a consensus of opinion from the thrombophilia specialist and pulmonologist, the warfarin was stopped. Two weeks later, he was admitted to the intensive care unit with mesenteric ischemia. A CT scan of the abdomen showed perfusion abnormality of the liver and multiple infarcts in the kidney and bowel suggesting embolic phenomenon. Subsequently, he developed multi-organ failure and passed away, seven years after his diagnosis of Stage IV lung adenocarcinoma.

## Discussion

Certain prognostic factors are predictive of survival in patients with non-small cell lung cancer (NSCLC). Good prognostic factors include early stage at diagnosis, good PS, no significant weight loss (not more than 5%), and female gender [3-4]. Our patient had a good PS upon presentation.

In the absence of a driver mutation, where a specific inhibitor is the first line of treatment, combination cytotoxic chemotherapy with a platinum-based doublet remains the backbone of the initial systemic treatment for patients with advanced NSCLC. Despite his Stage IVA disease, he received CRT, which is a deviation from the standard guidelines. This regimen was opted in view of his good PS and absence of extrathoracic disease. However, the intent was better disease control rather than definitive treatment.

After completion of chemotherapy, erlotinib was given as maintenance treatment. In the absence of disease progression, maintenance therapy with erlotinib after four cycles of platinum-based chemotherapy has been shown to prolong progression-free survival (PFS) compared to placebo, irrespective of EGFR status [5]. Later, when his disease progressed on erlotinib, we decided to give pemetrexed with bevacizumab as subsequent therapy. This combination regimen was studied in a Phase 2 trial showing a promising median PFS of four months, which was higher than previously studied single agents [6]. An acceptable alternative would have been to retreat the patient with the initial regimen, as the interval between treatment and disease progression was over six months [7].

Of note, both of the patient’s relapses occurred outside the thoracic cavity, suggesting that RT may have played a role in local disease control. Currently, we do not have a large body of literature to support the role of RT in M1a disease. The above case is an example suggesting the feasibility of CRT in M1a disease in the absence of extrathoracic disease. A similar case of M1a disease of squamous histology was treated with curative intent with neoadjuvant chemotherapy followed by surgery, another deviation from the standard guidelines [8]. In such circumstances, thinking outside the box may help prolong survival in selected patients.

In the past year, advances have been made in Stage IV NSCLC with the approval of novel agents, such as the combination of the vascular endothelial growth factor-2 inhibitor, ramucirumab, with docetaxel and the immune checkpoint inhibitors, nivolumab and pembrolizumab, as therapies in the relapsed setting. Our patient would have been appropriate for one of these treatments as well should his disease have progressed again. Unfortunately, he passed away from thromboembolic complications, an indirect consequence of malignancy. Nevertheless, he represents a case of exceptional response to therapy, surviving seven years with Stage IV NSCLC with good quality of life. 

## Conclusions

Stage IV lung cancer portends an extremely poor prognosis. However, long-term survival is possible in a small minority of patients. Identifying clinical and genetic prognostic indicators may help determine which patients will benefit from an aggressive multimodality therapeutic regimen as opposed to a palliative approach.
